# Epigenetic modifications in plant abiotic stress adaptation: towards climate-resilient and sustainable crop improvement

**DOI:** 10.3389/fpls.2026.1738299

**Published:** 2026-02-11

**Authors:** Muslim Qadir, Navjot Kaur, Faiz Ur Rahman, Farhan Nabi, Zienab F. R. Ahmed, Jian Wu

**Affiliations:** 1 College of Agriculture, South China Agricultural University (SCAU), Guangzhou, China; 2 Integrative Agriculture, College of Agriculture and Veterinary Medicine, United Arab Emirates University, Al Ain, United Arab Emirates

**Keywords:** abiotic stress adaptation, chromatin remodeling, climate-resilience crops, epiallele breeding, epigenomics, stress memory

## Abstract

Abiotic stresses such as drought, salinity, heat, and cold are the most critical factors limiting global crop productivity, posing significant challenges to food security and the sustainability of agricultural systems. Epigenetic modifications, including DNA methylation, histone modifications and non-coding RNAs, enable plants to respond rapidly to environmental stimuli without altering DNA sequences. These mechanisms, demonstrated through studies using whole-genome bisulfite sequencing (WGBS), ChIP-seq, ATAC-seq, and validation in key mutants (*met1*, *hda6*, *brahma*), mediate chromatin remodelers (*SWI*/*SNF*, DDM1), hormone signaling crosstalk, and emerging spatial epigenomics (scATAC-seq in roots and guard cells). This review synthesizes the hierarchy of somatic stress memory, characterized by sustained *H3K4me3* enrichment at promoters that facilitates rapid re-induction and transgenerational inheritance mediated by RdDM across the F_1_-F_3_ generations. By distinguishing correlative profiling from causal evidence, this review bridges significant experimental gaps, highlights the intricate, dynamic interplay between epigenetic layers that underpins stress memory and its heritable effects. Crop applications reveal the role of natural epialleles in promoting resilience: hypomethylation of *OsHMA3* promoter confers cadmium tolerance in rice grains (>50% reduction), while *DRO1* demethylation enhances drought adaptation over deeper rooting (15-22% yield protection). CRISPR-dCas9 epigenome editing enables targeted modifications, with *OsDREB1* targeting in rice boosting drought tolerance by 25% and TaNHX*1* modification in wheat developing salinity resilience. These advances position epigenetic regulation as a transformative tool for climate-resilient crop breeding. Integrating multi-omics with functional genomics addresses polyploid challenges, enabling non-transgenic epiallele breeding for global food security.

## 1 Introduction

Epigenetics, originally coined by Waddington to describe gene-environment interactions shaping phenotypes, now refers to heritable changes in gene expression that occur without alterations in the underlying DNA sequences (Waddington, 2012; Balard et al., 2024). In plants, DNA methylation, histone modifications, and noncoding RNAs together drive developmental plasticity and adaptation to abiotic stress by coordinating chromatin state with environmental cues (Abdulraheem et al., 2024). These mechanisms enable plants to integrate environmental cues with developmental programs, rapidly adjusting growth, flowering, and stress tolerance without genetic mutations (Hirayama and Shinozaki, 2010). Crops are subjected to abiotic stresses, such as drought, salinity, heat, cold, and nutrient deficiencies, which disrupt cellular homeostasis, increase reactive oxygen species (ROS), impair photosynthesis and threaten food security (Paes De Melo et al., 2022; Shafi et al., 2024).

This synthesis follows three logical pillars. Firstly, causal epigenetic mechanisms validated by WGBS, ChIP-seq, ATAC-seq and key mutants such as *met1*, *hda6*, *brahma* establish causality. Secondly, crop applications spanning roots and fruits under drought, heat and salt (*OsHMA3* Cd tolerance, tomato *HSP22* heat memory). Thirdly, memory hierarchy from somatic (*H3K4me3* retention) to transgenerational (*RdDM* F1-F3 inheritance). This framework eliminates redundancy while elucidating context-specific regulation (Iwasaki and Paszkowski, 2014).

Stress-induced epigenetic reprogramming activates stress-responsive genes and establishes somatic memory and transgenerational inheritance, priming progeny for recurrent challenges (Latzel et al., 2016; Zhao et al., 2019). Epigenetic memory refers to mitotically stable chromatin states enabling predictive adaptation to recurrent stresses. Somatic memory features *H3K4me3* retention at stress-responsive promoters, persisting post-drought to accelerate re-induction (Ding et al., 2012; Zheng et al., 2017). Transgenerational memory transmits *RdDM*-mediated silencing across F1-F3 generations (Iwasaki and Paszkowski, 2014; Lancíková et al., 2023). Unlike genetic variation, epigenetic diversity displayed as epialleles or epigenetic recombinant inbred lines (RILs), offers exploitable nongenetic variation for breeding climate-resilient crops, particularly perennials (Venios et al., 2024). CRISPR-dCas9 epigenome editing platforms employ catalytically dead Cas9 fused to epigenetic effectors (*TET1* for active demethylation, *DNMT3A* for targeted methylation) without DNA breaks, enabling precise chromatin manipulation. In Arabidopsis, dCas9-TET1 achieves targeted DNA demethylation at specific loci, demonstrating heritability across generations (Gallego-Bartolomé et al., 2018). dCas9 platforms enable site-specific gene activation in maize and stress-responsive pathways (Papikian et al., 2019). These tools establish causality between chromatin modifications and phenotypic outcomes (Mahdi Moradpour and Siti Nor, 2020). As the field progresses, challenges remain to efficiently translate these insights into practical applications across diverse plant species and agricultural systems (Colicchio et al., 2023).

Epigenomic profiling techniques, including Whole Genome Bisulfite Sequencing (WGBS) for DNA methylation, Chromatin Immunoprecipitation Sequencing (ChIP-seq) for *H3K4me3* and *H3K27me3*, and Assay for Transposase-Accessible Chromatin Sequencing (ATAC-seq) for chromatin accessibility, have identified key stress regulators. These mechanisms, validated in *Arabidopsis* mutants such as *met1* (TE derepression) and *hda6* (hypersensitivity) (Law and Jacobsen, 2010; Marand et al., 2021), are translatable to crops. Epialleles of *OsHMA3* reduce Cd accumulation in rice grains by 50%, while *OsDRO1* demethylation enhances drought tolerance, improving yield by 22% through deeper rooting (Gallego-Bartolomé et al., 2018). Mutant analyses and CRISPR perturbations further confirm the causality of these epigenetic modifications, supporting the potential of non-transgenic epiallele breeding to mitigate the projected 15-25% yield losses due to climate change (Iwasaki and Paszkowski, 2014; Huang and Jin, 2022).

This review synthesizes experimental evidence on how epigenetic mechanisms causally regulate crop responses to abiotic stress, with an emphasis on study designs that distinguish correlation from causation. By evaluating methodological rigor across studies, we identify key gaps, such as the limited causal data in crops, and highlight opportunities for integrating multi-omics with functional genomics to enhance crop improvement. The review addresses central questions, including how DNA methylation, histone modifications, and noncoding RNAs mediate stress-induced gene expression reprogramming; which experimental approaches (WGBS, ChIP-seq, ATAC-seq integrated with mutants/CRISPR) can establish causality in somatic and transgenerational stress memory; and how validated epigenetic targets can be harnessed for climate-resilient crop breeding.

## 2 Mechanisms of epigenomic modifications in plants

### 2.1 DNA methylation

DNA methylation at cytosine residues (CG, CHG, CHH contexts) is a primary epigenetic mechanism controlling gene expression and abiotic stress tolerance, mapped by WGBS (Leichter et al., 2022). DNA methyltransferases (*DRM2*, *MET1*, *CMT3*) maintain marks during replication, while demethylases enable dynamic responses. The *met1*/and *cmt3* mutants show stress sensitivity, confirming causal roles in silencing transposable elements (TEs) and stress-responsive genes (Rehman et al., 2022; Liu et al., 2023).

#### 2.1.1 DNA methylation and transposon silencing

**Figure 1:**
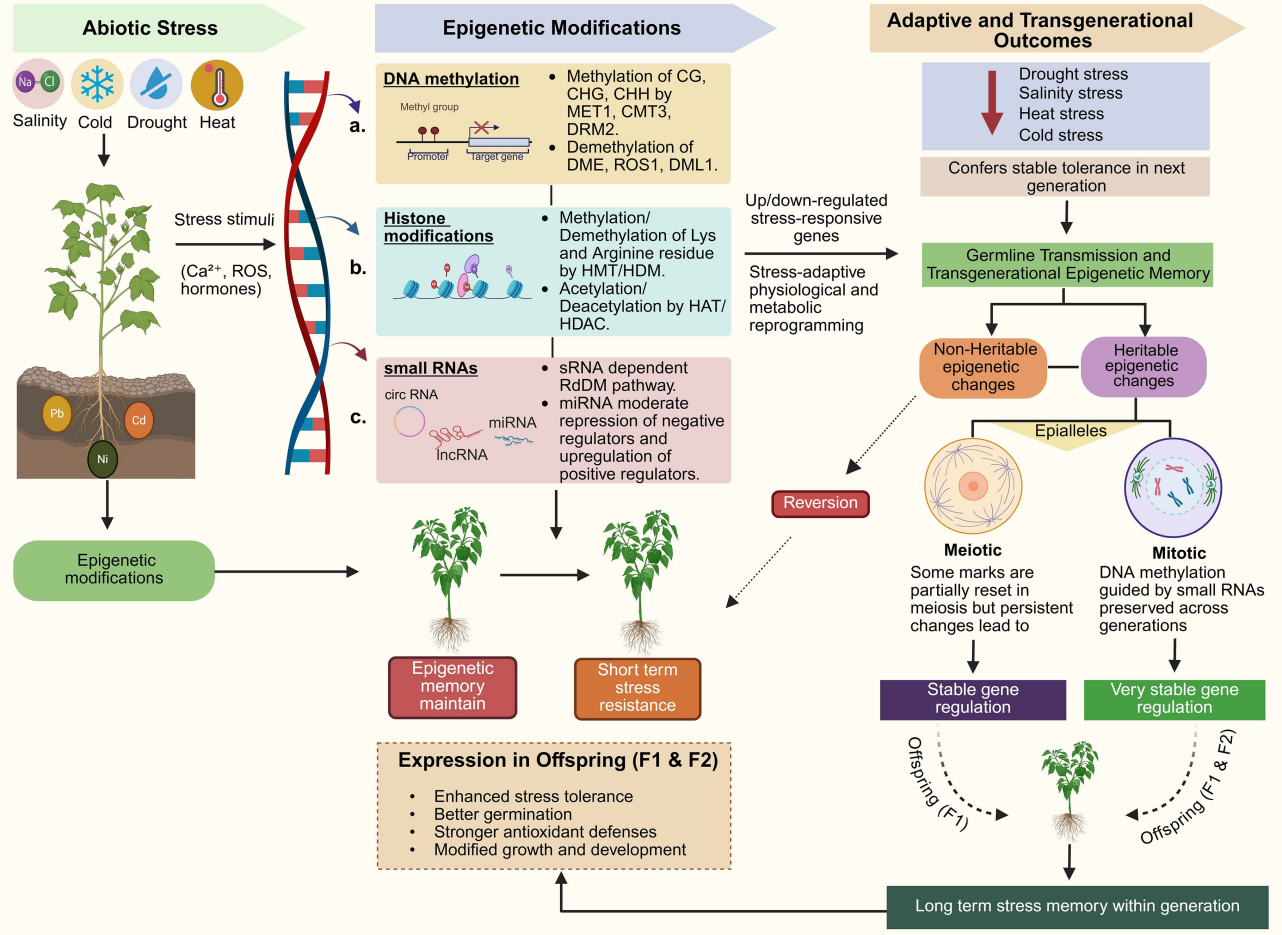
Epigenetic modifications and transgenerational inheritance of stress tolerance in plants under abiotic stress. This figure illustrates how abiotic stress induces epigenetic modifications, which regulate plant responses and contribute to stress memory. It highlights the role of non-heritable epigenetic changes that provide short-term stress resistance and heritable modifications passed down across generations by mitotic and meiotic inheritance. These heritable changes result in transgenerational epigenetic memory, conferring enhanced stress tolerance in offspring (F1 and F2). Further demonstrates how these modifications contribute to long-term adaptation and improved stress resilience in future generations.

DNA methylation regulates gene expression under abiotic stress conditions by regulating TE. Under abiotic stress, WGBS reveals TE hypomethylation that reactivates nearby stress-responsive genes, promoting phenotypic plasticity (Ramakrishnan et al., 2022). These patterns are illustrated in **Figure 1**, showing TE-gene interactions under stress. Stress-induced hypomethylation reactivates *Ty1* and *Ty3 retrotransposons* in Moso bamboo, producing lncRNAs and circRNAs that target stress genes, to correlative evidenced by bisulfite sequencing (Ding et al., 2024). These dynamic methylation changes contribute to both immediate responses and stress memory. However, causal validation requires TE-specific epigenome editing, which remains limited (Ma et al., 2024).

#### 2.1.2 Methylation patterns in stress-responsive genes

**Figure 2:**
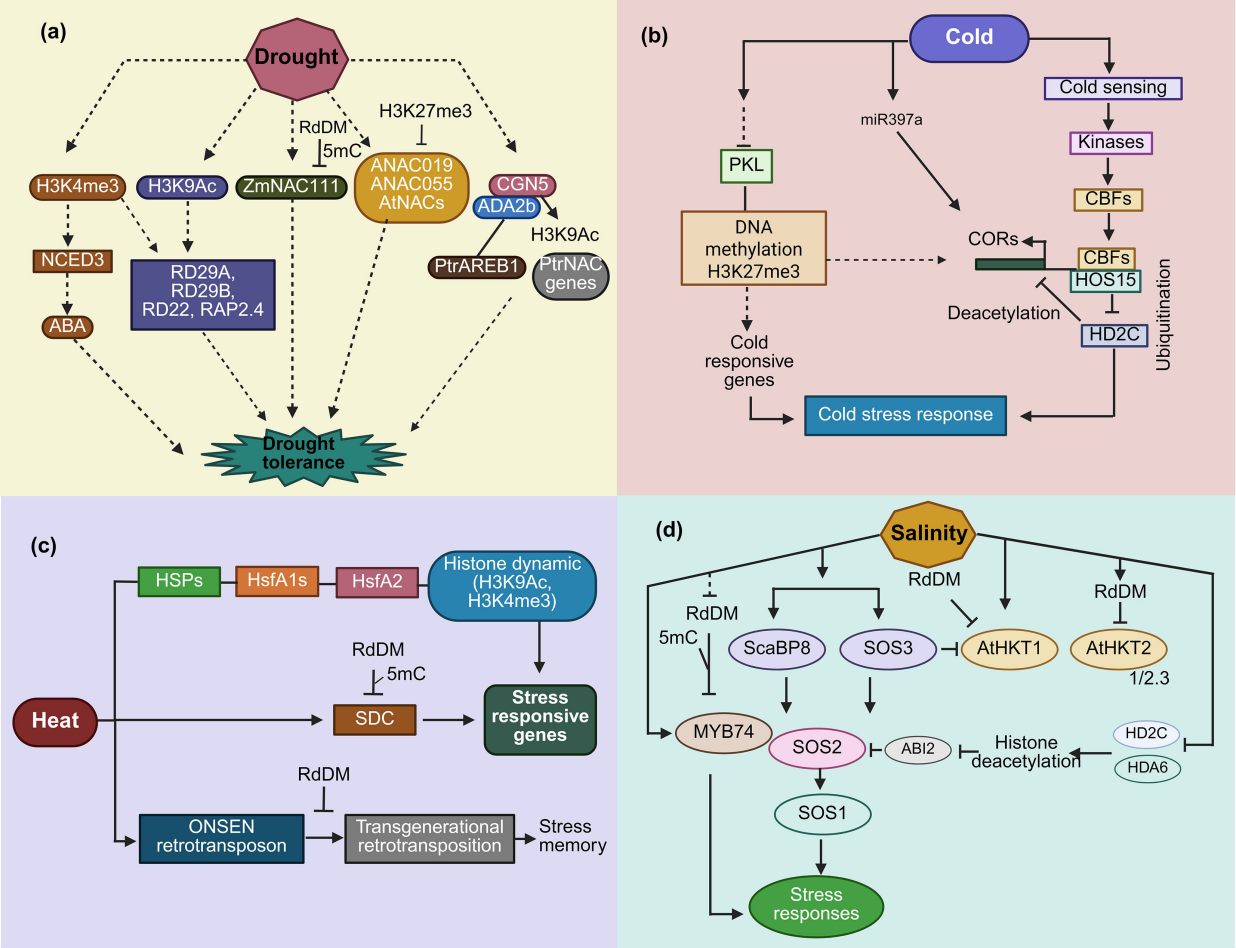
Comparative analysis of epigenetic regulation of stress-responsive genes in crop species under abiotic stresses. This figure compares the epigenetic regulation of stress-responsive genes varies across different abiotic stresses. **(a)** DNA methylation and RdDM regulate transposable elements in drought. **(b)** Cold stress triggers H3K27me3 and DNA methylation to repress gene expression by PKL and HD2C. **(c)** Heat stress activates H3K4me3 and H3K9Ac, and retrotransposon activation (ONSEN) contributes to transgenerational memory. **(d)** Salinity and drought stress involve RdDM and H3K4me3 at key stress-response loci like SOS1 and RD29A with HDA6-mediated deacetylation.

Environmental stress can also cause variations in methylation forms that affect the expression of stress-responsive and plant growth-regulating genes. In rice, bisulfite sequencing revealed promoter hypomethylation of *OsGLP8–10* and *OsGLP12* under salinity, which correlated with upregulation, though no functional mutants have been reported (Anum et al., 2023). Chickpea gene-body CG hypermethylation under salinity inversely correlates with expression (correlative; WGBS data), while foxtail millet demethylase upregulation suggests active adaptation (Gupta and Garg, 2023; Sun et al., 2024). DNA methylation thus modulates ABA signaling pathway genes during drought stress. **Figure 2** illustrates the epigenetic regulation of stress-responsive genes under various abiotic stresses, including drought, cold, heat, and salinity, highlighting key modifications such as DNA methylation, histone modifications, and small RNAs. Targeted demethylation through dCas9-TET1 could enable breeding (Yin et al., 2024). **Table 1** integrates DNA methylation with histone modifications discussed in Section 2.2, offering a summary of the key mechanisms involved.

**Table 1:** Epigenetic mechanisms and their roles in stress-responsive gene regulation across abiotic stresses in crop species.

Abiotic stress	Crops	Epigenetic modifications	Phenotypic effect	References
Drought	*Arabidopsis thaliana*	Overexpression of ScRIPK (RLC VII) is associated with stress signaling	Promotes flowering	Fang et al., 2024
	*Zea mays*	H3K4me3, H3K9ac, and H3K36me3 enrichment at stress-related loci	Improve the survival, anthesis and grain yield	Xu et al., 2017
	*Oryza sativa*	Site-specific DNA methylation changes in drought stress	Increase growth, proline and antioxidant activity	Kumar et al., 2023
	*Gossypium hirsutum*	Reduced H3K9ac level at GhWRKY33 promoter through GhHDT4D repression	Improves drought resistance and productivity	Zhang et al., 2020b
	*Arabidopsis thaliana*	Increased H3K9ac histone acetylation level at the promoter of 14 stress genes	Boosts drought tolerance	Zheng et al., 2016
	*Cicer arietinum*	Accumulation of miR408 transcripts	Increases leaf number and plant height	Hajyzadeh et al., 2015
	*Arabidopsis*	H4R3sme2-type histone methylation at the ANAC055 promoter	Higher proline levels and enhanced drought resistance	Fu et al., 2018
	*Dendrobium officinale*	Histone deacetylation mediated by a conserved family of 14 HDAC genes	Stimulates plant growth and development	Zhang et al., 2020b
	*Hordeum vulgare* and *Oryza sativa*	24-nt hc-siRNA–mediated RNA-directed DNA methylation at the HvCKX2.1 promoter	Quick shoot emergence in the next generation	Surdonja et al., 2017
	*Gossypium hirsutum*	Silencing GhHDT4D altered H3K9 histone acetylation, specifically increasing acetylation at the *GhWRKY33* locus	Improved growth and boll formation by reducing drought stress	Zhang et al., 2020a
Salinity	*Arabidopsis thaliana*	GCN5 acetylates H3K14 and H3K9 expression, and activating CTL1, MYB54 and PGX3 genes	Enhance growth under salt stress	Zheng et al., 2019
	*Zea mays*	RdDM (RNA-directed DNA methylation), which is mediated by KTF1	Improve salt resistance and biomass	Wang et al., 2025a
	*Arabidopsis thaliana*	m^6^A methylation reduces the stability of salt-responsive genes (SOS1, SAD1 and PIP1)	Increase germination and seedling vigor	Amara et al., 2022
	*Arabidopsis thaliana*	Multiple ABRE and G-box cis-acting elements which likely modulate gene expression	Boost phenotypic traits associated with enhanced salt tolerance	Shen et al., 2023
	*Zea mays*	rpd1-1/rmr6 epiregulator mutation, led to altered regulation of stress-related genes	Enhanced plant growth or sensitivity to salt stresses	Forestan et al., 2016
	*Zea mays*	DNA methylation caused loss of CHH methylation at many loci and reductions in CG and CHG methylation at specific loci	Stimulates plant growth	Li et al., 2014
Heat and Cold	*Arabidopsis thaliana*	HDA9 removes histone variation H2A.Z at YUC8 nucleosome, promoting thermomorphogenesis	Improve flowering	Van Der Woude et al., 2019
	*Brassica rapa subsp. pekinensis*	lncRNAs at the BrFLC2as locus cause epigenetic regulation	Stimulates flowering	Shea et al., 2019
	*Arabidopsis thaliana*	H3K4me3 and H3K4me2 elevation maintain heat stress memory-related genes	Increase survival and germination percentage	Friedrich et al., 2021
	*Arabidopsis thaliana*	HDA9 interacts with the PWR complex controls YUC8 by chromatin remodeling	Promotes overall plant growth	Shen et al., 2019
	*Beta vulgaris*	DNA hypomethylation in non-CpG contexts driven by the activation of demethylation pathways and modulate gene expression	Enhances the plant’s ability to adapt to fluctuating environmental conditions	Gutschker et al., 2022
	Arabidopsis	Histone deacetylation by HDA9, HDA15, and HDA19 with upregulation of temperature- and metabolism-related genes	Enhanced thermal response	Shen et al., 2019
	Arabidopsis, *Oryza sativa*	Expression of OsDREB1C/E/G genes may alter downstream ROS-scavenging and cell-death regulation pathways	Reduced chilling tolerance and heightened susceptibility to multiple abiotic stresses	Wang et al., 2022
	*Arabidopsis thaliana*	Histone deacetylation at the *YUCCA8* locus and eviction of the H2A.Z histone variant	Increased auxin biosynthesis and thermomorphogenic growth	Van Der Woude et al., 2019
	*Brassica rapa*	Long noncoding RNAs particularly natural antisense transcripts associated with vernalization-related genes BrFLC and BrMAF	Promotion of flowering through vernalization	Shea et al., 2019
	*Arabidopsis thaliana*	HDAC (histone deacetylase increases H3 acetylation and COR activation at the COR promoter	Increase survival and growth	Park et al., 2018
Heavy metals	*Hydrilla* *verticillata*	Hypermethylation induces DRM, SUVH6 and CMT expression	Enhance growth and biomass	Shi et al., 2017
	*Triticum aestivum*	DNA hypomethylation activates heavy metal ATPase2 and cassette metal detox genes	Promotes germination and root development	Shafiq et al., 2019
	*Arabidopsis thaliana*	DNA methylation changes in the AtPCR2 regulatory regions	Enhanced biomass, chlorophyll content, and heavy-metal tolerance	Reddy et al., 2023
	*Oryza sativa*	Triggered DNA methylation reprogramming and altered expression of methylation-modified genes	Improved seedling growth	Feng et al., 2016

### 2.2 Histone modifications in stress-responsive gene regulation

Histone modification as also known as post-translational alteration that dynamically restructure chromatin to regulate gene expression, enable dynamic modulation of stress-responsive genes under abiotic stress. These changes facilitate rapid adaptation to environmental cues and establish stress memory, enhancing long-term tolerance (Chwialkowska et al., 2016; Singh et al., 2021).

#### 2.2.1 Histone acetylation and deacetylation

Histone acetylation and deacetylation are crucial processes that govern the expression of genes by modulating the chromatin structure to enhance transcriptional activation of stress-responsive genes under abiotic circumstances (Wang et al., 2024). The histone acetyltransferases (HATs) are mainly responsible for the mechanisms of histone acetylation that involve the linking of an acetyl group to the lysine chain on the histone end, namely *H3K9* and *H3K14*. This modification stops this molecular protein from interacting with DNA and produces a more accessible, unstructured chromatin that encourages transcription, as it reduces the positive charge in histone. Rice salt stress increases *H3K9ac* and *H3K14ac* at *OsPP2C49* (ChIP-seq), enhancing tolerance with causal evidence from HAT overexpression lines (Liu et al., 2023). The function of histone acetylation in stress adaptation is demonstrated by the distinct transcriptional and metabolic behaviors shown in *Arabidopsis* plants that express the histone acetyltransferase *HAC1* from *Medicago truncatula* across salt and cold (Ivanova et al., 2023). In contrast, histone deacetylases (HDACs) regulate the expression of genes by eliminating acetyl groups. These cause chromatin condensation and transcriptional repression in adaptation to stress (Vincent et al., 2022). However, *HDA6* (Histone Deacetylase 6) and its homolog *HDA19* mediate stress-responsive deacetylation (Ueda and Seki, 2020). *H3K27ac*, a well-established activation mark at enhancers and promoters, differs from acetylation in gene bodies (Huang and Irish, 2024; Wang et al., 2025b). *hda6* exhibits jasmonate hypersensitivity and stress-related defects, confirming that *HDA6* plays a causal role in repressing stress genes through deacetylation, as demonstrated by ChIP-qPCR analyses (Chang et al., 2020; Vincent et al., 2022; Sun, 2025).

#### 2.2.2 Histone methylation and gene silencing

Histone methylation modifications on particular lysine residues can promote or inhibit gene expression. It contributes to the determination of memory of stress and controls metabolism, which in turn increases the resistance of plants to drought, salinity, and extreme temperatures (Shi et al., 2024). Trimethylation of H3 at lysine 4 (*H3K4me3*) is generally associated with transcriptional activation, whereas trimethylation at lysine 27 (*H3K27me3*) is associated to gene silencing. ChIP-seq reveals that *H3K27me3* represses cold-responsive genes before stress, with repression alleviated upon stress induction. This relationship is confirmed by *clf/swn* mutants, which affect *H3K27* methyltransferases (Wang et al., 2025b). These modifications are crucial for immediate stress responses and also for establishing epigenetic stress memory, which helps plants withstand recurring stresses (Jin et al., 2024). A specific histone modification genes that respond to various abiotic stresses has been identified in *Brassicaceae*, revealing their potential for developing stress-tolerant crops (Hu et al., 2023). Moreover, histone methylation regulates the expression of stress adaptive genes through interacting with other epigenetic processes such as DNA methylation and chromatin remodeling (Dar et al., 2022; Grgić et al., 2025). **Table 1** summarizes the regulation of histone modifications (*H3K4me3* activation and *H3K27me3* repression) across various abiotic stress contexts, integrating these with the effects of DNA methylation. In fruits, a critical agricultural organ, heat stress induces histone acetylation (*H3K9ac* and *H3K27ac*) at the promoters of HSPs in tomatoes (Kumar et al., 2021) and triggers dynamic chromatin remodeling in strawberries (López et al., 2024), similar to the responses observed in roots.

### 2.3 RNAs and non-coding RNAs

In order to influence the entire plant stress responses, small RNAs (miRNAs, siRNAs, and lncRNAs) regulate stress responses at transcriptional, post-transcriptional, and epigenetic levels via multilayered networks (Samynathan et al., 2023).

#### 2.3.1 Role of small RNAs (miRNAs, siRNAs)

In plants, the small non-coding RNAs (ncRNAs) mediate the regulatory response for the genes that respond to abiotic stress (**Figure 1**). These ncRNAs are involved in diverse regulatory interactions that determine how adaptable a plant might be to environmental stimuli. miRNAs (20–24 nucleotides; small RNA-seq + degradome) promote target mRNA degradation and inhibit translation, regulating heat shock proteins (HSPs) and NF-Y factors to maintain cellular homeostasis. Target mimics confirm the causal role of miRNAs in this process (Basso et al., 2019; Samat et al., 2024). Similarly, miRNAs actively participate in cross-kingdom interactions and may play a key role in influencing plant stress responses and agronomic traits through their interactions with other plants and microorganisms (Ding et al., 2024). siRNAs (lisiRNAs, nat-siRNAs; small RNA-seq) target and degrade stress-related mRNAs, facilitating adaptation. *AGO1/4* mutants exhibit defects in this process. These interactions suggest highly complex ncRNA regulatory networks that provide resilience to abiotic stress. Additionally, the regulation of non-coding RNAs regarding anthocyanin biosynthesis and their function as ROS scavengers and stress tolerance enhancers (Zhou et al., 2023).

#### 2.3.2 Long non-coding RNAs (lncRNAs)

RNA-seq and chromatin isolation revealed lncRNAs regulating chromatin remodeling, histone modification, and stress memory (Magar et al., 2024). lncRNAs act at multiple layers (RNA-seq + RIP-seq), associating with chromatin modifiers (ChIP assays) to alter DNA/histone marks (Hazra et al., 2023). lncRNAs employ various mechanisms to affect this role, most importantly, the alteration of chromatin structures, which adjusts the chromatin structure to be more or less condensed. lncRNAs perform this function through the association of chromatin-modifying proteins and agents that affect histone and DNA methylation changes.

For instance, lncRNAs help tether chromatin remodelers to precise genomic locations. This results in modifications to the structural chromatin that enable the activation of gene transcription during stressful conditions (Hazra et al., 2023). Hence, lncRNAs control the activation of which fundamental stress-responsive gene pathways are regulated by the phytohormones ABA and salicylic acid, both of which are crucial to the stress adaptation response in plants (Jin et al., 2024). ceRNA networks act as sponges for miRNAs that regulate stress-related genes, as revealed by RNA-seq and luciferase assays (Rakhi et al., 2024). Other regulatory lncRNAs might act as competing endogenous RNA (ceRNA) in concert with these miRNAs to influence the expression of a target mRNA (Xiao et al., 2022). For example, suitable lncRNAs, which act as ceRNAs for miRNAs and affect the expression of intricate genes involved in the stress response, have been reported in *Oryza sativa* (Rakhi et al., 2024). Similarly, lncRNAs and generic circRNAs interact with miRNAs via ceRNA networks that regulate important drought tolerance-associated genes, such as starch synthase 4 in switch grass (Guan et al., 2024). In addition, lncRNAs mediate the mechanisms through which plants develop stress memory to allow them to “remember” stressful events from the past and respond effectively to subsequent disturbances (Traubenik et al., 2024). This multifarious function of lncRNAs with regard to chromatin remodeling and stress responses raises much hope for using lncRNAs as targets in emerging crop varieties that can resist stress, in light of the avenues it opens up for agricultural innovation in climate change scenarios (Yang et al., 2023).

#### 2.3.3 Epitranscriptomic and RNA modifications

RNA modifications such as methylation and acetylation play a major role in regulating plant performance under abiotic stress. However, they run through an overall broader area of epitranscriptomics, which includes all chemical changes that occur among RNA molecules and modify the gene expression and adaptation of plants against various environmental stresses. m^6^A-seq revealed that RNA methylation regulates mRNA stability, splicing, and translation under stress (Cai et al., 2024). Such an epigenetic modification does not alter the DNA sequence itself. However, it can be inherited and has been proposed as a mechanism for transgenerational stress memory, enhancing plant resilience across generations (Essemine et al., 2025). This integration of modifications into breeding strategies holds a bright future in terms of developing stress-resistant crops, as it provides a method for enhancing adaptability without altering the genetics within plants (Rao et al., 2024). Such improvements in high-throughput sequencing skills have greatly contributed to identifying and understanding these modifications and their ability to improve crop yield and quality under adverse environmental conditions (Essemine et al., 2025). The elaborate interaction system, which emerges among important ncRNAs, such as miRNAs, siRNAs, and lncRNAs themselves, forms a highly sophisticated regulatory network for perceptive response-behavior patterns of plants against all possible environmental stresses for an improved capacity to endure and acclimate to threatening conditions (Yang et al., 2023).

#### 2.3.4 Chromatin remodeling, hormone crosstalk, and spatial epigenomics

Chromatin remodelers dynamically reposition nucleosomes at stress-responsive loci, facilitating rapid transcriptional responses. *SWI/SNF* complexes (*BRAHMA* subunit) and *DDM1* play essential roles in maintaining chromatin accessibility under abiotic stress, as demonstrated by ATAC-seq profiling. The *brahma* mutants exhibit severe drought sensitivity, with reduced root growth and impaired gene activation, confirming the complex causal involvement in ABA-responsive loci as ChIP-seq *H3K4me3* (Venkatesh and Workman, 2015). *DDM1* prevents TEs reactivation under salinity stress, and *ddm1* mutants show genome instability and salt hypersensitivity, with WGBS and RNA-seq providing evidence of context-specific hypomethylation (Pecinka et al., 2010; Zemach et al., 2013). *ISWI* remodelers, such as *CHR3*, regulate flowering time under heat stress, with *chr3* mutants exhibiting early flowering (Kang et al., 2022).

Hormonal signaling pathways, including ABA, JA and auxin, which are intricately linked with epigenetic regulation to coordinate stress responses. The ABA signaling pathway requires *H3K27me3* demethylation by *JMJ30/KDM4C* at *ABI5* and *RAB18* promoters. *AtJmj30* mutants show ABA insensitivity and increased drought susceptibility, further supporting the epigenetic regulation of ABA responses (ChIP-seq) (Ay et al., 2009; Yamaguchi et al., 2021). Jasmonic acid (JA) signaling recruits *HDA6* to deacetylate JAZ repressors and stress-related genes, with *hda6* mutants displaying defects in jasmonate-mediated defense and stress crosstalk (ChIP-qPCR) (Liu et al., 2010; Chang et al., 2020). Auxin homeostasis is repressed by *RdDM* under drought stress through promoter hypermethylation of *GH3* auxin genes (WGBS); ros1 demethylase mutants show ectopic auxin accumulation and reduced stress tolerance (Yu et al., 2013; Hayashi et al., 2021). Ethylene response factors (ERFs) require *H3K36* demethylation by *SDG8* for heat stress tolerance, with *sdg8* mutants showing enhanced heat tolerance (ChIP-seq) (Chang et al., 2020).

Tissue-specific epigenetic regulation is revealed through spatial epigenomics techniques. Single-cell ATAC-seq (scATAC-seq) in roots shows drought-induced changes in chromatin accessibility, specifically in the stele versus cortex, correlating with aquaporin expression. This finding is validated by scWGBS (Ma et al., 2025). Guard cell bisulfite sequencing reveals CG hypomethylation linked to stomatal aperture regulation, supported by live-cell imaging and WGBS (Siqueira et al., 2021). Long-read nanopore sequencing enables the resolution of polyploid crop epialleles, such as wheat homologs, identifying subgenome-specific stress marks through *ONT* WGBS (Angeloni et al., 2022). The meristem-specific scRNA-seq and scATAC-seq analyses have uncovered progenitor-specific *H3K27me3* bivalency, enabling rapid cold acclimation (Theler et al., 2014).

Integration of chromatin remodelers (*SWI, SNF and DDM1*), hormone signaling (ABA, JA and auxin), and spatial epigenomics (scATAC and WGBS) reveals the convergence of these mechanisms at stress hubs. The functional validation through *brahma*, *hda6*, *ros1* and *jmj30* mutants establishes causality beyond correlative relationships. Moreover, epiallele editing using dCas9-TET1 at *SWI/SNF* loci improves rice drought tolerance (Gallego-Bartolomé et al., 2018), and dCas9-KRAB in wheat enhances salinity resilience (Mccarty et al., 2020).

## 3 Mechanism of epigenetic memory in stress response

Epigenetic memory in plants enables heritable modification of gene expression without DNA sequence changes, allowing sessile organisms to anticipate recurrent abiotic stresses (drought, heat, salinity). DNA methylation, histone modifications, and RNA-directed pathways transmit these marks across generations (Rai et al., 2023; Ahtisham and Obaid, 2024). This phenotypic plasticity enhances resilience to future challenges (Sun et al., 2021). An example is stress priming, when crops are exposed to mild stress, which can result in both temporary and sustained physiological and molecular changes, which might be inherited by the plant in the future (Lagiotis et al., 2023). This memory not only helps to respond to stress instantly but also prepares the next generations to face similar challenges, which makes the plant phenotypically plastic and able to survive in various environments (Ashapkin et al., 2020). Their knowledge of the mechanisms is critical to the development of strategies of crop improvement to improve stress tolerance, which becomes more and more significant in the context of climate change and the necessity of sustainable agriculture (Chinnusamy et al., 2013). Although epigenetic memory is functional during the lifetime of individual plants, there is increasing evidence that epigenetic marks induced by stress can be transmitted across generations, thus modifying adaptive behaviors in the generation of offspring in transgenerational epigenetic inheritance **Figure 1**.

### 3.1 Transgenerational epigenetic inheritance

The development of abiotic stress resilient crops requires the knowledge of the mechanism of how epigenetic alterations are transmitted across generations. Priming or memory is also regarded as an essential part of these epigenetic modifications, contributing to the enhanced capacity to endure stress in the future, even without being primed by the same stress (Kumar and Rani, 2023). However, priming is not always observed, as it can influence the growth and development of plants. **Table 2** summarizes transgenerational epigenetic effects across various stresses, revealing both transient and stable epi-alleles (Crisp et al., 2016; Mozgova et al., 2019).

**Table 2:** Epigenetic regulatory networks involved in stress memory and transgenerational inheritance in plants.

Stress	Epigenetic mechanism	Primary contact and treatment	Transgenerational response	Model plant	Reference
Drought, salt and heat	DNA methylation	Priming of first-generation plants with drought, heat and osmotic stress and strains	Drought-induced memory loss enhanced tolerance to heat, drought and salt stress	Wheat	Wang et al., 2016; Zhang et al., 2016; Tabassum et al., 2017
Drought and salt	lncRNAs	Osmotic stress	Improved abiotic stress response through epigenetic alterations	Maize	Forestan et al., 2016
Cold	–	H_2_O_2_ root pretreatment and fruit heat exposure from arginase induction	Antioxidant activation, reduction of chilling injury and enhanced oxidative stress ability	Tomato	İşeri et al., 2013; Zhang et al., 2013
Heavy metal	–	Low temperature acclimation	Cold-induced photo inhibition	Pea	Streb et al., 2008
Cold, heat and drought	DNA methylation	Cold acclimation	Increase heat and drought resistance with enhanced growth and yield	Canola	Liu et al., 2017; Hatzig et al., 2018
Drought	–	Osmopriming and drought stress	Boosted growth under water stress	Alfalfa	Mouradi et al., 2016
Heat and cold shock	small RNAs	Heat, drought and salinity	Stress-induced cross-protection and transgenerational inheritance	Field mustard and Turnip	Bilichak et al., 2015; Kellenberger et al., 2018

Stress triggers the formation of epigenetic stress memory in plants in the form of epi-alleles that can be either transient or permanent (Molinier et al., 2006). This consistent memory following stress is maintained throughout the plant’s development cycles or transmitted to successive generations, leading to plant adaptability and evolution. However, it is possible to reverse transient memory when the stress is removed. Plants enter the germline in late development, they memorize the stress they face in life, and they most likely do it through epigenetic processes in the lineage of cells which form the germ-line and transmit it to the generations (Lang-Mladek et al., 2010). For example, drought stress in Arabidopsis has been shown to result in histone demethylation at the promoters of specific genes, leading to increased expression and improved tolerance to future drought (Shafi et al., 2024). More recently, epigenetic regulation through DNA methylation is one component of transgenerational memory in rice under heavy metal stress. After heavy metal was removed, the memory of expression developed, and heavy metal-transporting P-type ATPase genes (HMAs) were activated due to heavy metal-associated stress (Cong et al., 2019). **Figure 2** illustrates the epigenetic regulation of stress-responsive genes across abiotic stresses. It highlights how DNA methylation, histone modifications, and small RNAs regulate key genes such as *SOS1*, *RD29A* and *ONSEN* in response to drought, cold, heat, and salinity stress (Kang et al., 2022). *H3K4me3* was also enriched on the coding region of submergence-inducible genes in rice plants subjected to a water submergence environment, and a reduction in *H3K4me2* was observed. These histone modifications were temporarily restored to normal levels upon re-aeration (Tsuji et al., 2006). These histone modifications may not be inherited by subsequent generations, as they cause temporary epi-alleles that diminish upon stress removal. In another study, it was found that when Arabidopsis plants were exposed to temperature stress, transcriptional gene silencing was released at many heterochromatin sites. This destabilized condition was confirmed by transcriptome analysis at the genome level (Pecinka et al., 2010). The effect of this transcriptional activation was temporary and repression occurred upon a few days of stress elimination. Recently, DNA replication-linked modification of the H3.1 histone variant has been shown to replace the transcriptional repressive label *H3K27me3* in daughter plant cells (Jiang and Berger, 2017). Most of the epigenetic changes induced by stress are temporary and reversible upon stress removal. However, some modifications are irreversible and can be transmitted through mitotic or meiotic divisions (**Figure 1**).

### 3.2 Molecular pathways involved in epigenetic memory

Epigenetic memory in plants is primarily mediated by DNA methylation and histone modifications; among these, cytosine DNA methylation stabilizes the genome while regulating stress-responsive gene expression (Liu and He, 2020). These marks establish somatic memory across mitotic divisions and are transmitted meiotically to progeny (**Figure 2**). Histone H3 methylation at K27 and K4 is equally critical in the processes underlying the memory of stress, K27 and K4 methylation predispose cells to defense mechanisms and assist quick adaptation to stress by reorganizing chromatin and altering the accessibility of certain genes (Zheng et al., 2022). It is dynamic and reversible, as plants usually respond to environmental cues and modulate gene expression. This is robust phenotypic plasticity and resilience (Kumari et al., 2022).

The interplay of histone modifications and DNA methylation with non-coding RNAs constitutes a complex system of regulation that allows plants to adjust to varying surroundings, even with restrictions on the genome. This is important for the evolution of plants and for breeding purposes to develop resilient crops (Kumar and Mohapatra, 2024). In addition, epigenetic marks in plants are reset through rejuvenation, revealing the flexibility and plasticity of such mechanisms and showing that they are essential for maintaining genomic integrity and facilitating developmental transitions. CRISPR-dCas9 epigenome editing targets *H3K27me3* (KRAB domain), DNA methylation (DNMT3A fusion), and *H3K4me3* for precise epigenetic memory modification. Rice drought tolerance improves by dCas9-TET1 targeting stress-responsive loci, while wheat salinity resilience is enhanced through dCas9-KRAB at *SWI/SNF* promoters (Gallego-Bartolomé et al., 2018; Mccarty et al., 2020).

## 4 Applications of epigenomic modifications in crop improvement

### 4.1 Epigenetic markers for crop breeding

Epigenetic markers offer nongenetic variation for breeding climate-resilient crops, enabling rapid adaptation without altering the DNA sequence. For example, natural epialleles at the *OsHMA3* promoter reduce cadmium accumulation in rice grains by >50% through vacuolar sequestration, demonstrating how promoter CG hypomethylation can serve as both a biomarker and direct breeding target for heavy metal tolerance (Sasaki et al., 2014). Similarly, *DRO1* promoter demethylation variants enhance deep rooting under drought, correlating with 15-20% yield gains in field trials (Sun et al., 2021). DNA methylation thus silences transposable elements while activating stress-responsive loci like *CBF3* and *SOS1*, as validated by WGBS across tolerant genotypes (Gupta and Garg, 2023). Histone modifications provide additional markers; *ATX1 H3K4me3* enrichment at NHX antiporters distinguishes salt-tolerant wheat lines (Ding et al., 2024). lncRNAs such as *CRIR1* modulate cold-responsive methylation, suggesting utility for marker-assisted selection in cereals (Li et al., 2024). **Table 1** integrates these epigenetic markers (DNA methylation, histone modifications, and non-coding RNAs) and their applications in breeding. Combining these epialleles with SNPs through dual genetic-epigenetic selection accelerates the development of stress-resilient cultivars (Ma et al., 2024).

Epigenetics-assisted breeding technology is increasingly geared toward improving crops, thereby utilizing the epigenetic landscape to enhance plant traits and stress resistance. CRISPR/Cas-based epigenome edits have been used for epibreeding to improve maize yield and yield stress responses (Zhang et al., 2024b). In addition, controlling epigenetic modifications suggests the potential regulation of phenotypic expression that the fruit ripening and tuberization in tomatoes and potatoes entail, hence, creating more possibilities in breeding strategies (Zhang et al., 2024b). The combination of genome-editing tools, such as CRISPR-Cas9, permits the accurate manipulation of epigenetic traits to improve climate-resilient crops by enhancing thermotolerance and other stress adaptations (Qi et al., 2023). Epigenetic markers used for targeted changes to seed traits in Camelina sativa provide more ways for seed size and oil quality to be refined (Turcotte, 2024). Moreover, the study of epigenetic control of wheat and other cereal breeding for resistance to abiotic stress has also revealed the intricacy of these control mechanisms (Saripalli et al., 2023). The incorporation of epigenetic mechanisms in breeding has been facilitated more by the advanced sequencing of the genome, deepening our understanding of the epigenome and epitranscriptome, for new pathways in crop improvement (Yung et al., 2023). The intricate relationships of differing epigenetic modifications provide for the coherence, consistency, and robustness of the control mechanisms.

### 4.2 Epigenetic reprogramming in crop improvement

The mechanisms of epigenetic reprogramming shape how stress memories are passed down between generations of various crops. These mechanisms alter and silence the expression of certain genes (Gallusci et al., 2023). These phenomena enhance the ability of plants to withstand abiotic stress. Further, it enables more efficient memory of the stress to improve generation upon generation. Stress priming involves exposing plants to mild stress. This results in physiological and molecular alterations in plants that can persist throughout a plant life span and be passed down to offspring. This can lead to improved stress tolerance in subsequent generations (Lagiotis et al., 2023). This transgenerational reminiscence provides plants with another important adaptation strategy for responding to rapidly changing environments: the ability to cope with the re-emergence of stress (Ahtisham and Obaid, 2024). The noteworthy role of epigenetic mechanisms is the ability to engender permanent, yet flexible, alterations in gene expression that may be exploited to bolster resilient and productive crops (Villagómez-Aranda et al., 2022). The principles of seed priming, wherein stress exposure before germination enhances stress tolerance and germination, provide further evidence of the phenomenon across generations (Louis et al., 2023). The leverage such breeding strategies could provide is immense, fostering a viable pathway for the development of crops that endure climatic stresses while eliminating the use of chemical methods (Mladenov et al., 2021). The reliability, heritability, and permanence of epigenetic markers pose challenges for such strategies, which may result in the alterations being ephemeral and not stably transmitted to subsequent generations (Jin et al., 2024). The intricacy of potential epigenetically engendered consequences is worth investigating, misguided gene expression. Employing transgenerational stress memory for epigenetic reprogramming opens exciting avenues for advancements in agriculture but necessitates careful monitoring and management of the relevant epigenetic elements to guarantee that the anticipated benefits are not overshadowed by the potential risks.

### 4.3 CRISPR-Cas9 and epigenome editing

CRISPR-Cas9 epigenome editing enables the precise modification of epigenetic marks without DNA cleavage, including *H3K27me3* repression (KRAB domain fusion), DNA demethylation (TET1), *H3K4me3* activation, and *H3K9ac* deposition (*GCN5*). Drought tolerance in rice improves by 25% through dCas9-TET1 targeting the *OsDREB1* promoters, while wheat exhibits enhanced salinity resilience through dCas9-KRAB at the *TaNHX1* loci (Gallego-Bartolomé et al., 2018; Papikian et al., 2019). CRISPR-Cas9 technology is increasingly being employed to modify the epigenomes subjected to enhance abiotic stress tolerance and improve environmental resilience in crops. Considering the CRISPR-Cas9 systems are exceedingly efficient, accurate, cost-effective and have gained broad acceptance as an appropriate method for developing high-performing, stress-resistant crop varieties (Furrow, 2017). Gene-editing has enhanced the silencing of genes that play key roles in stress reaction pathways that regulate activities such as antioxidant, osmotic regulation, and heat shock proteins, which are broadly involved in nature’s work on abiotic stress tolerance (Doggalli et al., 2025). CRISPR-Cas9s are further availed for transcribing resistance and making epigenetic alterations that are beneficial for plant behavior under abiotic stress (Alvares et al., 2024). Epigenetic alterations have been demonstrated to improve tolerance and flexibility in adjusting to various conditions by influencing the expression of genes involved in stress response (Papikian et al., 2019; Chang et al., 2020). With the help of CRISPR-Cas9, it became possible to develop stress-resilient and high-yielding rice varieties by targeting *OsF3H-1* and *OsCHS31* genes involved in drought response and yield potential (Shafana et al., 2025). Identically, CRISPR-Cas9 editing techniques targeted at increasing resilience in legumes will enhance their yield potential and increase the likelihood of withstanding stress (Singh et al., 2024). This technology can enhance crop performance without foreign DNA incorporation, presenting itself as a viable alternative to classical breeding strategies, fastened by lengthy protocols and the natural occurrence of genetic variation (Habibi, 2024). While the issues of regulatory restrictions and off-target effects are growing, CRISPR-Cas technologies are improving, particularly with new forms of prime and base editing designed to enhance precision and control (Chen et al., 2024). This reinforces the reality of using global population pressures and associated challenges regarding food sustainability and security (Niraula et al., 2024).

## 5 Epigenomic modifications in crops under abiotic stresses

### 5.1 Drought stress

Drought significantly reduces crop productivity by impairing photosynthesis, inducing stomatal closure, and altering root architecture (Seleiman et al., 2021; Shafi et al., 2024). WGBS profiling in maize, wheat, barley, cotton and mung bean reveals locus-specific hypomethylation, which activates genes involved in osmolyte synthesis (P5CS), ROS scavenging, and ABA biosynthesis (Chwialkowska et al., 2016; Zhu, 2016; Wang et al., 2021; Naderi et al., 2024). In rice, promoter demethylation at drought-responsive loci has been observed, and met1 mutants have been used to confirm the causal role of DNA methylation (Liang et al., 2014; Virlouvet and Fromm, 2015).

Histone modifications play a key role in coordinating ABA signaling. ChIP-seq analysis identifies *H3K36me3* enrichment at the *OsNCED3* and *OsNCED5* promoters, mediated by *SDG708*, with *sdg708* mutants displaying increased drought sensitivity (Chen et al., 2021). Additionally, the BES1-TPL-HDA19 complex deacetylates *ABI3* chromatin, reducing ABA sensitivity, and *hda19* mutants exhibit ABA hypersensitivity (Ryu et al., 2014). *Brassica napus* exhibits *H3K4me3* gain and *H3K27me3* loss at proline synthesis loci, indicating a role in drought tolerance (Prasad et al. (2024). m^6^A-seq shows m^6^A stabilization of drought mRNAs in sugarcane and foxtail millet with the *SiYTH1* reader binding to *CSK* (Luo et al., 2023; Wei et al., 2023). In cotton, *GhALKBH10B* demethylase destabilizes ABA and Ca²^+^ signaling transcripts, with *ghalkbh10b* mutants showing enhanced drought tolerance (Li et al., 2023). In maize, circRNAs in roots are associated with *H3K36me3* and *H3K4me1* marks, suggesting a role in regulating drought response (ChIP-seq) (Xu et al., 2024).

#### 5.1.1 Epigenetic regulation in drought-tolerant varieties

Tolerant genotypes exhibit controlled epigenomic plasticity, distinguishing them from sensitive varieties across crops (Sun, 2025)WGBS/BS-seq; (Yang et al., 2025). Maize and cotton tolerant lines exhibit stable global methylation, with targeted promoter hypomethylation at ABA biosynthesis (*NCED3*), ROS scavenging (SOD and CAT) and osmoprotectant synthesis (P5CS), enabling the rapid activation of stress-responsive genes. In contrast, sensitive genotypes display erratic genome-wide hyper- and hypomethylation patterns that correlate with poor acclimation and yield loss, offering potential for actionable epiallele selection (Zhang et al., 2024a).

ChIP-seq profiling reveals that *H3K4me3* and *H3K9ac* are rapidly established at osmoprotectant and *LEA* gene promoters within hours in drought-tolerant maize, sorghum and cotton, while sensitive lines show delayed or absent chromatin opening (Prasad et al. (2024). *miR169g* represses NF-YA transcription factors more efficiently in tolerant varieties, leading to reduced stomatal aperture and enhanced water conservation, as validated by degradome analysis and transgenics (Rao et al., 2024). Ca²^+^ and Na^+^ fluxes trigger scATAC-seq identified open chromatin specific to root responses in tolerant genotypes, highlighting ion-epigenome crosstalk (Miryeganeh, 2025). These epigenomic patterns provide deployable epialleles for marker-assisted selection.

#### 5.1.2 Epigenetic modifications in root development

Root system architecture (RSA) plasticity is a key target for epigenetic engineering aimed at enhancing drought avoidance in field conditions (Nguyen et al., 2022). WGBS reveals ABA-responsive promoter demethylation at the *DRO1* (deep rooting) and *PLT1* (lateral root density) loci, which control adaptive RSA phenotypes. *Dro1* mutants exhibit defective deep rooting, highlighting the role of this loci in root plasticity (Sun et al., 2022). In barley, root tissues show organ-specific hypermethylation, with stele tissues exhibiting elevated CG methylation compared to the cortex under water deficit conditions. ChIP-seq analysis confirms the exclusion of *H3K27me3* from aquaporin and hydraulic conductivity genes, linking chromatin modifications to root function under drought stress (Chwialkowska et al., 2016).

Hormonal signaling pathways, including ethylene and JA, amplify epigenetic regulation of RSA. These pathways require *H3K36me3* deposition by *SDG8* at root hair development and suberization genes. *SdG8* mutants produce shallow, poorly suberized roots, underscoring the importance of *H3K36me3* in root adaptation (Ranjan et al., 2022; Gao et al., 2025). Wheat QTL-methylation overlap analyses across diverse panels have identified breeding targets, linking natural epiallele variation to RSA ideotypes. These epialleles confer a 15-20% yield advantage under terminal drought conditions (Siddiqui et al., 2023). Single-cell ATAC-seq reveals compartment-specific changes in chromatin accessibility, coordinating the responses of the stele, cortex and endodermis. **Figure 2** shows the multi-layered coordination of DNA, histone modifications, and non-coding RNAs (ncRNAs) in driving adaptive RSA under progressive water deficit. These findings position epigenetic markers as complementary tools for classical RSA QTL breeding.

### 5.2 Temperature stress

#### 5.2.1 Cold stress regulation

The C-repeat binding factor-cold-regulated (CBF-COR) pathway demonstrates the epigenetic regulation of cold acclimation (ChIP-seq/ATAC-seq) (Kwon et al., 2009). Cold exposure triggers the dynamic removal of *H3K27me3* from *COR15A*, *RD29A* and *LEA* promoter regions through the antagonism of Polycomb Repressive Complex 2 (*PRC2*) with *clf* and *swn* double mutants exhibiting cold hypersensitivity. The *PKL* and *SWR1* chromatin remodeling complex mediates RdDM-independent activation at *CBF* and *DREB1* loci, with *pkl* mutants showing hypersensitivity to cold stress (Yang et al., 2019; Carter et al., 2018). The PWR-HOS15-HD2C complex deposits H4 acetylation at COR promoters during cold acclimation, and hd2c mutants exhibit defects in freezing tolerance (Lim et al., 2020).

Histone deacetylase *HDA6* is transcriptionally induced by cold, establishing basal repression that allows for rapid de-repression upon stress, with *hda6* mutants showing increased sensitivity to cold (To et al., 2011; Park et al., 2018). Long non-coding RNA (lncRNA) *CRIR1* recruits *DRM2* demethylase to alter cold-responsive methylation patterns in cassava, as confirmed by WGBS validation (Li et al., 2024). The *SWI* and *SNF* chromatin remodeler, specifically the *LFR* subunit, activates transcription at the *CBF3* and *ICE1* loci, with *lfr* mutants showing defects in cold response (Ma et al., 2023). Histone variant H2A.Z eviction, accompanied by *H3K4me3* gain, occurs at cold memory loci, enabling recurrent cold tolerance (Grgić et al., 2025). These epigenetic modifications contribute to the establishment of cold memory and stress resilience (**Table 1**).

#### 5.2.2 Heat stress and epigenetic regulation of heat shock proteins

The transcriptional memory of heat shock proteins (HSPs) is governed by persistent chromatin marks as demonstrated by ChIP-seq and MNase-seq analyses (Pratx et al., 2023; Arıkan et al., 2025). The continuous enrichment of *H3K4me3*, coupled with low nucleosome turnover at the promoters of *HSP22*, *HSP70*, and *sHSP*, enables rapid re-induction during recurrent heat waves, contributing to developed thermotolerance. *JUMONJI* C-domain demethylases (*JMJ30* and *32*) actively remove repressive *H3K27me3* from HSP promoters, with *jmj* mutants showing impaired heat acclimation (Yamaguchi et al., 2021, Yamaguchi et al., 2020). In wheat, hypomethylation of the *HSP17.6* promoter correlates with stronger heat induction across genotypes, as revealed by WGBS profiling (El-Shehawi, 2020).

In barley, DNA demethylation integrates with *H3K9ac* deposition at heat-responsive loci, a process confirmed by ChIP-seq (Li et al., 2022). *miR398* targets Cu and Zn-SOD, and *miR156* targets *HSFA2*, regulating ROS detoxification and heat shock factor amplification, as demonstrated by degradome analysis and transgenic studies (Meiri and Breiman, 2009; Samanta and Thakur, 2015). *ONSEN* retrotransposon-derived siRNAs enhance *HSF1* binding and prevent genome instability (Cavrak et al., 2014). The *H3K36me3* reader EBS plays a critical role in maintaining HSP memory across cell divisions. In maize, genome-wide changes in *H3K4me2* and *H3K9ac* are accompanied by *HsfA2* binding, further illustrating the coordinated role of chromatin and non-coding RNA regulation in heat stress response (Friedrich et al., 2021). This coordinated chromatin-ncRNA regulation establishes thermos-memory, which can be leveraged for breeding heat-resilient varieties in response to climate warming.

### 5.3 Salinity stress

Salinity stress reduces the crop growth, yield and quality. Salinity stress occurs due to an overabundance of Na and Cl soil ion accumulations, which significantly interfere with processes like photosynthesis, nutrient absorption and hormonal balances (Balasubramaniam et al., 2023). Salt stress also directly causes osmotic and ionic imbalances, oxidative stress, tissue damage, and even death in a plant (Hasanuzzaman, 2020). Cell Na^+^ ions and cytosolic Ca^2+^ stimulate the formation of hyperactive Ca^2+^ and the silencing of the *SOS3-SOS2* module pathway. This alters the *SOS1* (Na^+^/H^+^ antiporter) phosphorylation and activity, and also targets the *Arabidopsis* AKT1-K^+^ channel, H^+^ ATPase, and putative Mg^2+^ transporter (Zhu, 2016). The salt stress response in root systems, including the roles of *SOS1* and *HKT1* ion transporters, is illustrated in **Figure 1**. This figure highlights how the ion transport activity of *SOS1* is regulated by DNA methylation and salt tolerance-promoting histone modifications. The ion transport activity of *SOS1* is refined with DNA methylation and salt tolerance-promoting histone modifications. *HKT1* provides salt tolerance by limiting Na^+^ influx and acts as a counterbalance to the SOS pathway (Rus et al., 2001). Salt stress in the mangrove *Bruguiera gymnorhiza* is characterized by the hypermethylation of roots, especially of transposable elements (Miryeganeh et al., 2022).

In maize, salt stress enhances the expression of stress-responsive genes, including *ZmXET1* and *ZmEXPB2*, which correlates with increased levels of *H3K9* acetylation. In contrast, wheat demonstrates a GCN5-mediated activation of salt tolerance genes through acetylation (Roy and Soni, 2022). These modifications allow for the dynamic adjustment of root systems, which improves salinity resilience and overall productivity.

#### 5.3.1 Salt-induced DNA methylation

The whole-genome bisulfite sequencing (WGBS) conducted across chickpea, wheat, and rice has identified hypomethylation at the *SOS1* and *HKT1;5* promoters as a key feature of salt-tolerant genotypes. The accumulation of toxic Na^+^ in *HKT1;5* mutants further underscores the importance of these epigenetic modifications in salt tolerance (Gupta and Garg, 2023; Baek et al., 2011). Histone acetyltransferase *GCN5* is involved in mediating *H3K9* and *H3K14* acetylation at cell wall remodeling genes such as *PGX3*, *CTL1* and *MYB54*, with *gcn5* mutants showing increased sensitivity to salt stress (Zheng et al., 2019). In soybeans, salt priming induces global hypomethylation that activates ABA-dependent *LEA* and osmolyte genes, thereby providing cross-protection (Boyko and Kovalchuk, 2011; Yung et al., 2024).

In maize, the transcription factor *ZmKTF1* directs RdDM-mediated CHH methylation at oxidoreductase loci in response to salinity (Law and Jacobsen, 2010; Wang et al., 2025a). **Figure 2** illustrates *RdDM*-TE silencing across stresses, with heat-induced *ONSEN* transgenerational activation making heritable stress memory (Kang et al., 2022). m^6^A-seq analysis reveals that *FIONA1* and *MTA* methyltransferases stabilize aquaporin *PIP1* and *SOS1* mRNAs, with *fio1* mutants displaying impaired stabilization (Cai et al., 2024). Altered DNA methylation patterns at salt-resilience loci are observed in wild wheat introgression lines, highlighting the role of epiallele flow during breeding (Hoseini et al., 2024). In rice, *OsBISAMT1* facilitates salt-induced RNA m6A modifications at stress-responsive transcripts (Amara et al., 2022). These multi-omics datasets collectively reveal deployable epialleles that link DNA methylation dynamics to ion homeostasis, osmoprotection, and hormonal signaling, offering valuable targets for marker-assisted selection of salt-tolerant cultivars.

#### 5.3.2 Histone modifications and salt stress response

Dynamic histone modifications play a crucial role in reshaping chromatin at genes involved in the salinity response, as demonstrated by ChIP-seq analysis (Shi et al., 2024). Enrichment of *H3K4me3* at ion transporters (NHX1), ROS scavengers (*APX2*), and transcription factors (WRKY) characterizes salt-tolerant genotypes, while repressive *H3K27me3* and *H3K9me2* decline enables activation (Chen and Wu, 2010; Wan et al., 2024). In rice, the histone deacetylase *OsHDA706* targets *H4K5* and *H4K5* deacetylation at the *OsPP2C49* promoter, integrating ABA signaling with salt response. The *oshda706* mutant exhibits defective salt stress responses (Liu et al., 2023).

In soybean, NF-Y transcription factor complexes recruit *H3K9ac* to salt-responsive genes, while the GmHDA13-GmFVE complex maintains repression under non-stress conditions, as validated by ChIP-qPCR (Lu et al., 2021). The histone variant H2A.Z modulates transcriptional flexibility at stress loci through thermosensitive deposition and eviction (Miao et al., 2024). Overexpression of Arabidopsis *HAC1* reprograms the transcriptome and metabolome under salinity, upregulating proline and polyamine biosynthesis pathways (Ivanova et al., 2023). *Trithorax ATX1* deposits *H3K4me3* at NHX antiporters, with *atx1* mutants showing impaired salt tolerance (Ding et al., 2011). The dynamic balance between active and repressive histone modifications, coordinated with DNA methylation and m^6^A RNA modifications, drives variation in genotypic tolerance. **Table 1** synthesizes these mechanisms for potential breeding applications.

### 5.4 Heavy metal stress

Heavy metals such (cadmium, lead, mercury, chromium, and arsenic) disrupt enzyme function, induce ROS bursts, and contaminate food chains, necessitating precise epigenetic regulation of detoxification pathways as WGBS/ChIP-seq/RNA-seq (Mahawar et al., 2023). The global hypermethylation silences TEs, while locus-specific promoter hypomethylation activates key transporters and chelators. For example, *OsHMA3* and *oszip1* mutants accumulate toxic cadmium in grains, highlighting the role of epigenetic modifications in metal tolerance (Liu et al., 2019). *miR166*, *miR164* and *miR390* target ZIP, NAC and auxin transcription factors, providing post-transcriptional control of metal exclusion as validated by degradome analysis across cereals (Chakrabarty, 2024). Enrichment of *H3K9ac* and *H3K4me3* at *GST*, *P5CS* and phytochelatin loci correlates with metal detoxification, with *hda6* and *hda19* mutants exhibiting hypersensitivity and increased metal accumulation (Niekerk et al., 2021). Dynamic expression cycles of *MET1* and *DRM2* coordinate waves of hyper- and hypomethylation in *Amaranthus* under chromium stress (Lancíková et al., 2023). Transgenerational inheritance of F2 generations in rice demonstrates stable epigenetic memory at tolerance loci, with epialleles being passed down through generations (Cong et al., 2019, Cong et al., 2024). These epialleles co-segregate with exclusion QTLs, facilitating dual genetic and epigenetic breeding strategies for developing metal-safe staple crops (**Table 1**), which summarizes deployable epigenetic markers for breeding applications.

#### 5.4.1 Epigenomic control of metal detoxification

Tonoplast sequestration serves as a prime example of high-value epigenetic engineering targets. In rice, overexpression of *OsHMA3* reduces Cd accumulation in grains by 10-fold through vacuolar compartmentalization, coordinated with ZIP-family transporter regulation under combined Zn, Cu and Cd stress. *Oshma3* mutants, however, are defective in root sequestration (Sasaki et al., 2014). WGBS profiling reveals that hypomethylation of the *OsZIP1* promoter specifically excludes Cd, Cu and Zn from shoots, while maintaining essential nutrient levels. Oszip1 mutants, on the other hand, enhance metal uptake (Liu et al., 2019).

Cd stress induces *H3K9ac* and *H3K4me3* deposition at key detoxification loci, including phytochelatin synthase *PCS1*, glutathione S-transferase GST, and metallothionein genes, as validated by ChIP-seq (Guarino et al., 2024). The histone deacetylases *HDA6* and *HDA19* maintain basal repression, enabling rapid activation upon metal exposure; *hda6* single and double mutants exhibit increased metal accumulation (Niekerk et al., 2021). *miR390* and *miR393* target *TIR1* and *AFB2* auxin receptors, modulating root exclusion and lateral root suppression, as demonstrated by degradome analysis and transgenic studies (Niekerk et al., 2021). scATAC-seq identifies chromatin opening at *NRAMP* and *IRT1* influx loci, specifically in salt-tolerant genotypes, highlighting epigenetic regulation of metal influx (Wang et al., 2025b). These findings suggest breeding targets across legumes, cereals, and brassicas, with potential for a 20% improvement in grain safety.

#### 5.4.2 Stress memory and breeding applications

Transgenerational epigenetic memory in rice persists across 1 to 3 generations after exposure to Cd or mercury (Hg) with heritable hypomethylation at the *OsHMA4* and *PCS2* detoxification loci enhancing tolerance in progeny, as tracked by WGBS inheritance profiling (Cong et al., 2019, Cong et al., 2024). This memory resets in clean soil after the F3 generation, balancing adaptation with plasticity (Iqbal et al., 2024). QTL-methylation overlap analyses across diverse rice and wheat panels have linked *MET1* and *DRM2* variants to Cd exclusion phenotypes, with potential for a 15-25% improvement in grain safety (Sharma et al., 2025). CRISPR-dCas9 targeting of *OsZIP1* and *OsHMA3* promoters enables reversible editing of metal exclusion traits without genetic load (Feng et al., 2023). Wild rice and wheat introgressions carry pre-adapted methylation states at metal homeostasis loci, offering a foundation for breeding (Hoseini et al., 2024).

Locus-specific siRNAs maintain silencing of TEs across F2 populations, as demonstrated by *RdDM* validation (Lancíková et al., 2023). m^6^A modifications, mediated by *FIONA1* and *MTA* complexes, stabilize detoxification mRNAs transgenerationally, enhancing stress resilience (Cai et al., 2024). Inheritance of *H3K27me3* at *NRAMP5* reinforces metal exclusion, contributing to salt tolerance (Ding et al., 2011). **Figure 1** demonstrates the integrated DNA-histone-ncRNA memory circuit. Dual genetic-epigenetic selection schemes position epialleles as powerful breeding tools for developing metal-safe cultivars.

## 6 Challenges and future perceptions

### 6.1 Current research limitations

The inherent complexity of epigenetic regulation represents the primary barrier to translating findings into crop breeding applications. The layered interactions between DNA methylation, histone modifications, and non-coding RNAs (ncRNAs) produce context-dependent effects, complicating the attribution of simple causal relationships. Multi-omics dissection, including WGBS, ChIP-seq, and small RNA-seq, remains essential but resource-intensive. **Table 2** provides an overview of the multi-omics integration workflow for epigenetic breeding applications, summarizing how these technologies can be combined to enhance crop breeding strategies. Current research predominantly relies on correlative profiling, with thousands of studies documenting stress-induced marks that correlate with tolerance phenotypes. However, causal validation requires more sophisticated approaches, such as the use of *met1*, *hda6*, or *ros1* mutants, or dCas9-based epigenome editing techniques that are still in the early stages of development, particularly for polyploid crops like wheat and maize.

Transient modification dynamics also pose significant challenges. Stress-induced hypomethylation or *H3K4me3* gains often revert after stress recovery, undermining the stable inheritance of epialleles necessary for breeding. Further complicating strategies are developmental programming variables, such as seasonal timing, stress dosage, and developmental stage, which contribute to epigenetic heterogeneity and complicate the implementation of universal approaches. The demand for high-resolution mapping tools, such as scATAC-seq for chromatin accessibility and nanopore WGBS for polyploid subgenomes, is constrained by substantial cost and bioinformatics barriers, particularly for understudied crops. The over-reliance on model systems, especially Arabidopsis, limits the translatability of findings, as polyploid genome architectures and perennial growth cycles in crops necessitate crop-specific validation.

### 6.2 Technological advancements and translation roadmap

Recent advances in single-cell epigenomics are revolutionizing the resolution of epigenetic landscapes, surpassing bulk assays. scATAC-seq and scWGBS distinguish methylation dynamics between the root stele and cortex during drought, identifying aquaporin regulators that remain undetectable in tissue averages (Ma et al., 2025). Long-read sequencing technologies, including PacBio HiFi and Oxford Nanopore, enable the assembly of repetitive polyploid genomes, while directly detecting m^6^A epitranscriptomic marks and large structural variants that are missed by short-read approaches (Liu et al., 2023). **Table 2** summarizes these multi-omics integration strategies, providing a roadmap for breeding applications. Chromatin accessibility assays, such as ATAC-seq, DNase-seq and CUT and RUN, systematically map regulatory elements under combined drought and heat stress conditions, revealing transcription factor binding networks. CRISPR-dCas9 multiplex screening accelerates epiallele discovery by systematically perturbing *H3K27me3* or DNA methylation at candidate loci. Nanopore direct RNA-seq captures the dynamic links between the epitranscriptome and stress, while spatial transcriptomics (MERFISH) correlates 3D chromatin organization with gene expression.

Translation platforms that integrate WGBS, scRNA-seq, and phenomics are advancing predictive epiallele-to-phenotype modeling. Machine learning frameworks, trained on integrated epigenomic datasets, have demonstrated the ability to predict yield under stress, with field-validated wheat dCas9 lines showing 15-22% gains in salinity tolerance without transgene integration (Liu et al., 2024). The minor crops such as millets, quinoa, and tef benefit disproportionately from cost-effective nanopore sequencing platforms, democratizing access to high-resolution epigenomics (Sahoo et al., 2020).

### 6.3 Ethical, ecological and regulatory considerations

Epigenetic engineering presents the potential for non-transgenic stress tolerance with minimal genomic alteration, yet it requires rigorous oversight. Off-target effects risk unintended consequences, including disruptions to the microbiome or soil microbial communities, necessitating long-term ecosystem monitoring that spans decades. Public perception remains a significant challenge, as consumers often conflate reversible epigenetic modifications with permanent genetic modifications, despite the fundamental mechanistic differences. This confusion is exacerbated by regulatory ambiguity, as current frameworks lack specificity for epigenetic editing.

Biodiversity concerns must also be considered, as the introduction of superior epialleles could result in the competitive displacement of wild relatives, thereby threatening genetic diversity. Additionally, potential disruptions to food webs, such as shifts in pollinator preferences and soil microbe composition, require longitudinal studies. Intergenerational tracking technologies are essential to verify the transient nature of beneficial epigenetic modifications without creating persistent ecological legacies. A responsible deployment framework should integrate comprehensive risk assessments (including off-target profiling and ecosystem modeling), prioritize equitable global access, particularly in food-insecure regions, and establish adaptive regulations that distinguish epigenetic editing from conventional breeding. International consortia should coordinate field trials, public communication efforts, and policy harmonization to ensure that epigenetic agriculture delivers climate resilience without compromising sustainability.

## 7 Conclusion

Epigenomic modifications such as DNA methylation, histone modifications, and non-coding RNAs are crucial for regulating crop responses to abiotic stresses. These mechanisms, validated through WGBS, ChIP-seq, ATAC-seq, and key mutants (*met1*, *hda6*, *brahma*), integrate chromatin remodelers (*SWI/SNF*, *DDM1*) with hormone signaling (ABA, JA and auxin) and spatial epigenomics (single-cell ATAC-seq). They establish somatic stress memory by sustained *H3K4me3* enrichment. The transgenerational inheritance occurs through RdDM-mediated silencing across F_1_-F_3_ generations. Field-validated epialleles (*OsHMA3* for Cd tolerance, *DRO1* for drought resistance) and CRISPR-dCas9 editing (*OsDREB1* rice 25% drought tolerance; *TaNHX1* wheat salinity resilience) demonstrate translational potential. Multi-omics integration addresses polyploid complexity, enabling non-transgenic breeding. These advances position epigenetics as a practical, scalable platform for climate-resilient crops under combined drought, salinity, heat and cold stresses, ensuring sustainable productivity and global food security.
